# Cyclophosphamide mechanism of action in preclinical tecemotide studies

**DOI:** 10.1186/2051-1426-1-S1-P75

**Published:** 2013-11-07

**Authors:** Ken Hance, Robert Tighe, Jin Qi, William Hastings, Guozhong Qin, Bo Marelli, Hong Wang, Huakui Yu, Yanping Zhang, Xiaomei Xu, Wilson Guzman, Masie Wong, Giorgio Kradjian, Beatrice Brunkhorst, Helen Sabzevari, Yan Lan, Robert Hofmeister, Michael Wolf

**Affiliations:** 1 ImmunoOncology, EMD Serono Research & Development Institute Inc., Billerica, MA, USA; 2 Merck KGaA, Darmstadt, Germany

## Background

Tecemotide is a MUC1 antigen-specific therapeutic cancer vaccine. In phase III clinical studies, delivery of tecemotide is preceded by a single low dose of cyclophosphamide (CPA) to inhibit regulatory T cells (Tregs) and enhance the response to the tumor-associated antigen. Here, we investigated effects of CPA on the immune environment and tumor growth in preclinical murine models.

## Methods

The effect of CPA was investigated in tumor-free human MUC1 transgenic mice and anti-tumor responses were evaluated in the mice engrafted with syngeneic colorectal and ovarian cancer cells expressing human MUC1. The mice received either vehicle control, CPA (100 mg/kg), tecemotide (100 μg) or CPA + tecemotide. The immune cell phenotype and function in the spleen were assessed on Days 1, 3 and 7 post-administration.

## Results

The single administration of CPA led to a reduction of the absolute numbers of splenocytes, including CD8^+^ cells and Tregs (p<0.05 on Days 1, 3 and 7 vs. control). But while by Day 7 post-administration the total number of splenocytes and CD8^+^ T cells partially recovered, the number of Tregs stayed low. This resulted in a higher CD8^+^/Treg ratio. Further, Tregs exhibited significantly reduced suppressor activity on a per cell basis as observed on Day 3. In the MC38/MUC1 colorectal cancer model, CPA alone reduced the growth of tumors, which was enhanced by the combination of CPA + tecemotide (2-way ANOVA, p<0.05). Tecemotide monotherapy and vehicle control did not show an effect. Likewise, mice treated with the combination survived longer (median survival 31 d vs. 19 d with tecemotide; 24.5 d, CPA; 19.5 d, control; P_TREND_=0.04). The combination therapy also significantly increased the precursor frequency of endogenous P15E-antigen-specific CD8^+^ T cells and IFN-γ production indicating antigen spreading. In the MUC1^+^ orthotopic ovarian cancer model MOSEC/MUC1, tecemotide inhibited tumor growth (2-way ANOVA, p<0.05) and the development of abdominal ascites. Tecemotide significantly elevated BP25-specific CD4^+^ T cell IFN-γ production as compared to the control or CPA, a response that was significantly enhanced by the combination of tecemotide with CPA.

## Conclusions

Besides its direct cytotoxic effects on tumor cells, CPA can reduce immunosuppressive mechanisms in the tumor and thereby create a favorable immune environment for the combination with tecemotide to exert its activity. These findings provide mechanistic evidence to support the use of low-dose CPA as a preconditioning agent designed to potentiate the immune and antitumor efficacy of tecemotide.

